# Measurement of the forward energy flow in *pp* collisions at $\sqrt{s}=7\ \mbox{TeV}$

**DOI:** 10.1140/epjc/s10052-013-2421-y

**Published:** 2013-05-17

**Authors:** The LHCb Collaboration, R. Aaij, C. Abellan Beteta, A. Adametz, B. Adeva, M. Adinolfi, C. Adrover, A. Affolder, Z. Ajaltouni, J. Albrecht, F. Alessio, M. Alexander, S. Ali, G. Alkhazov, P. Alvarez Cartelle, A. A. Alves, S. Amato, Y. Amhis, L. Anderlini, J. Anderson, R. B. Appleby, O. Aquines Gutierrez, F. Archilli, A. Artamonov, M. Artuso, E. Aslanides, G. Auriemma, S. Bachmann, J. J. Back, C. Baesso, V. Balagura, W. Baldini, R. J. Barlow, C. Barschel, S. Barsuk, W. Barter, A. Bates, Th. Bauer, A. Bay, J. Beddow, I. Bediaga, S. Belogurov, K. Belous, I. Belyaev, E. Ben-Haim, M. Benayoun, G. Bencivenni, S. Benson, J. Benton, A. Berezhnoy, R. Bernet, M.-O. Bettler, M. van Beuzekom, A. Bien, S. Bifani, T. Bird, A. Bizzeti, P. M. Bjørnstad, T. Blake, F. Blanc, C. Blanks, J. Blouw, S. Blusk, A. Bobrov, V. Bocci, A. Bondar, N. Bondar, W. Bonivento, S. Borghi, A. Borgia, T. J. V. Bowcock, C. Bozzi, T. Brambach, J. van den Brand, J. Bressieux, D. Brett, M. Britsch, T. Britton, N. H. Brook, H. Brown, A. Büchler-Germann, I. Burducea, A. Bursche, J. Buytaert, S. Cadeddu, O. Callot, M. Calvi, M. Calvo Gomez, A. Camboni, P. Campana, A. Carbone, G. Carboni, R. Cardinale, A. Cardini, H. Carranza-Mejia, L. Carson, K. Carvalho Akiba, G. Casse, M. Cattaneo, Ch. Cauet, M. Charles, Ph. Charpentier, P. Chen, N. Chiapolini, M. Chrzaszcz, K. Ciba, X. Cid Vidal, G. Ciezarek, P. E. L. Clarke, M. Clemencic, H. V. Cliff, J. Closier, C. Coca, V. Coco, J. Cogan, E. Cogneras, P. Collins, A. Comerma-Montells, A. Contu, A. Cook, M. Coombes, G. Corti, B. Couturier, G. A. Cowan, D. C. Craik, S. Cunliffe, R. Currie, C. D’Ambrosio, P. David, P. N. Y. David, I. De Bonis, K. De Bruyn, S. De Capua, M. De Cian, J. M. De Miranda, L. De Paula, P. De Simone, D. Decamp, M. Deckenhoff, H. Degaudenzi, L. Del Buono, C. Deplano, D. Derkach, O. Deschamps, F. Dettori, A. Di Canto, J. Dickens, H. Dijkstra, P. Diniz Batista, M. Dogaru, F. Domingo Bonal, S. Donleavy, F. Dordei, A. Dosil Suárez, D. Dossett, A. Dovbnya, F. Dupertuis, R. Dzhelyadin, A. Dziurda, A. Dzyuba, S. Easo, U. Egede, V. Egorychev, S. Eidelman, D. van Eijk, S. Eisenhardt, U. Eitschberger, R. Ekelhof, L. Eklund, I. El Rifai, Ch. Elsasser, D. Elsby, A. Falabella, C. Färber, G. Fardell, C. Farinelli, S. Farry, V. Fave, D. Ferguson, V. Fernandez Albor, F. Ferreira Rodrigues, M. Ferro-Luzzi, S. Filippov, M. Fiore, C. Fitzpatrick, M. Fontana, F. Fontanelli, R. Forty, O. Francisco, M. Frank, C. Frei, M. Frosini, S. Furcas, A. Gallas Torreira, D. Galli, M. Gandelman, P. Gandini, Y. Gao, J-C. Garnier, J. Garofoli, P. Garosi, J. Garra Tico, L. Garrido, C. Gaspar, R. Gauld, E. Gersabeck, M. Gersabeck, T. Gershon, Ph. Ghez, V. Gibson, V. V. Gligorov, C. Göbel, D. Golubkov, A. Golutvin, A. Gomes, H. Gordon, M. Grabalosa Gándara, R. Graciani Diaz, L. A. Granado Cardoso, E. Graugés, G. Graziani, A. Grecu, E. Greening, S. Gregson, O. Grünberg, B. Gui, E. Gushchin, Yu. Guz, T. Gys, C. Hadjivasiliou, G. Haefeli, C. Haen, S. C. Haines, S. Hall, T. Hampson, S. Hansmann-Menzemer, N. Harnew, S. T. Harnew, J. Harrison, P. F. Harrison, T. Hartmann, J. He, V. Heijne, K. Hennessy, P. Henrard, J. A. Hernando Morata, E. van Herwijnen, E. Hicks, D. Hill, M. Hoballah, P. Hopchev, W. Hulsbergen, P. Hunt, T. Huse, N. Hussain, D. Hutchcroft, D. Hynds, V. Iakovenko, P. Ilten, J. Imong, R. Jacobsson, A. Jaeger, M. Jahjah Hussein, E. Jans, F. Jansen, P. Jaton, B. Jean-Marie, F. Jing, M. John, D. Johnson, C. R. Jones, B. Jost, M. Kaballo, S. Kandybei, M. Karacson, T. M. Karbach, I. R. Kenyon, U. Kerzel, T. Ketel, A. Keune, B. Khanji, Y. M. Kim, O. Kochebina, I. Komarov, R. F. Koopman, P. Koppenburg, M. Korolev, A. Kozlinskiy, L. Kravchuk, K. Kreplin, M. Kreps, G. Krocker, P. Krokovny, F. Kruse, M. Kucharczyk, V. Kudryavtsev, T. Kvaratskheliya, V. N. La Thi, D. Lacarrere, G. Lafferty, A. Lai, D. Lambert, R. W. Lambert, E. Lanciotti, G. Lanfranchi, C. Langenbruch, T. Latham, C. Lazzeroni, R. Le Gac, J. van Leerdam, J.-P. Lees, R. Lefèvre, A. Leflat, J. Lefrançois, O. Leroy, Y. Li, L. Li Gioi, M. Liles, R. Lindner, C. Linn, B. Liu, G. Liu, J. von Loeben, J. H. Lopes, E. Lopez Asamar, N. Lopez-March, H. Lu, J. Luisier, H. Luo, A. Mac Raighne, F. Machefert, I. V. Machikhiliyan, F. Maciuc, O. Maev, S. Malde, G. Manca, G. Mancinelli, N. Mangiafave, U. Marconi, R. Märki, J. Marks, G. Martellotti, A. Martens, L. Martin, A. Martín Sánchez, M. Martinelli, D. Martinez Santos, D. Martins Tostes, A. Massafferri, R. Matev, Z. Mathe, C. Matteuzzi, M. Matveev, E. Maurice, A. Mazurov, J. McCarthy, G. McGregor, R. McNulty, F. Meier, M. Meissner, M. Merk, J. Merkel, D. A. Milanes, M.-N. Minard, J. Molina Rodriguez, S. Monteil, D. Moran, P. Morawski, R. Mountain, I. Mous, F. Muheim, K. Müller, R. Muresan, B. Muryn, B. Muster, J. Mylroie-Smith, P. Naik, T. Nakada, R. Nandakumar, I. Nasteva, M. Needham, N. Neufeld, A. D. Nguyen, T. D. Nguyen, C. Nguyen-Mau, M. Nicol, V. Niess, R. Niet, N. Nikitin, T. Nikodem, A. Nomerotski, A. Novoselov, A. Oblakowska-Mucha, V. Obraztsov, S. Oggero, S. Ogilvy, O. Okhrimenko, R. Oldeman, M. Orlandea, J. M. Otalora Goicochea, P. Owen, B. K. Pal, A. Palano, M. Palutan, J. Panman, A. Papanestis, M. Pappagallo, C. Parkes, C. J. Parkinson, G. Passaleva, G. D. Patel, M. Patel, G. N. Patrick, C. Patrignani, C. Pavel-Nicorescu, A. Pazos Alvarez, A. Pellegrino, G. Penso, M. Pepe Altarelli, S. Perazzini, D. L. Perego, E. Perez Trigo, A. Pérez-Calero Yzquierdo, P. Perret, M. Perrin-Terrin, G. Pessina, K. Petridis, A. Petrolini, A. Phan, E. Picatoste Olloqui, B. Pie Valls, B. Pietrzyk, T. Pilař, D. Pinci, S. Playfer, M. Plo Casasus, F. Polci, G. Polok, A. Poluektov, E. Polycarpo, D. Popov, B. Popovici, C. Potterat, A. Powell, J. Prisciandaro, V. Pugatch, A. Puig Navarro, W. Qian, J. H. Rademacker, B. Rakotomiaramanana, M. S. Rangel, I. Raniuk, N. Rauschmayr, G. Raven, S. Redford, M. M. Reid, A. C. dos Reis, S. Ricciardi, A. Richards, K. Rinnert, V. Rives Molina, D. A. Roa Romero, P. Robbe, E. Rodrigues, P. Rodriguez Perez, G. J. Rogers, S. Roiser, V. Romanovsky, A. Romero Vidal, J. Rouvinet, T. Ruf, H. Ruiz, G. Sabatino, J. J. Saborido Silva, N. Sagidova, P. Sail, B. Saitta, C. Salzmann, B. Sanmartin Sedes, M. Sannino, R. Santacesaria, C. Santamarina Rios, R. Santinelli, E. Santovetti, M. Sapunov, A. Sarti, C. Satriano, A. Satta, M. Savrie, D. Savrina, P. Schaack, M. Schiller, H. Schindler, S. Schleich, M. Schlupp, M. Schmelling, B. Schmidt, O. Schneider, A. Schopper, M.-H. Schune, R. Schwemmer, B. Sciascia, A. Sciubba, M. Seco, A. Semennikov, K. Senderowska, I. Sepp, N. Serra, J. Serrano, P. Seyfert, M. Shapkin, I. Shapoval, P. Shatalov, Y. Shcheglov, T. Shears, L. Shekhtman, O. Shevchenko, V. Shevchenko, A. Shires, R. Silva Coutinho, T. Skwarnicki, N. A. Smith, E. Smith, M. Smith, K. Sobczak, F. J. P. Soler, F. Soomro, D. Souza, B. Souza De Paula, B. Spaan, A. Sparkes, P. Spradlin, F. Stagni, S. Stahl, O. Steinkamp, S. Stoica, S. Stone, B. Storaci, M. Straticiuc, U. Straumann, V. K. Subbiah, S. Swientek, V. Syropoulos, M. Szczekowski, P. Szczypka, T. Szumlak, S. T’Jampens, M. Teklishyn, E. Teodorescu, F. Teubert, C. Thomas, E. Thomas, J. van Tilburg, V. Tisserand, M. Tobin, S. Tolk, D. Tonelli, S. Topp-Joergensen, N. Torr, E. Tournefier, S. Tourneur, M. T. Tran, M. Tresch, A. Tsaregorodtsev, P. Tsopelas, N. Tuning, M. Ubeda Garcia, A. Ukleja, D. Urner, U. Uwer, V. Vagnoni, G. Valenti, R. Vazquez Gomez, P. Vazquez Regueiro, S. Vecchi, J. J. Velthuis, M. Veltri, G. Veneziano, M. Vesterinen, B. Viaud, I. Videau, D. Vieira, X. Vilasis-Cardona, J. Visniakov, A. Vollhardt, D. Volyanskyy, D. Voong, A. Vorobyev, V. Vorobyev, C. Voß, H. Voss, R. Waldi, R. Wallace, S. Wandernoth, J. Wang, D. R. Ward, N. K. Watson, A. D. Webber, D. Websdale, M. Whitehead, J. Wicht, D. Wiedner, L. Wiggers, G. Wilkinson, M. P. Williams, M. Williams, F. F. Wilson, J. Wishahi, M. Witek, W. Witzeling, S. A. Wotton, S. Wright, S. Wu, K. Wyllie, Y. Xie, F. Xing, Z. Xing, Z. Yang, R. Young, X. Yuan, O. Yushchenko, M. Zangoli, M. Zavertyaev, F. Zhang, L. Zhang, W. C. Zhang, Y. Zhang, A. Zhelezov, A. Zhokhov, L. Zhong, A. Zvyagin

**Affiliations:** 1 CERN, 1211 Geneva 23, Switzerland; 2 Centro Brasileiro de Pesquisas Físicas (CBPF), Rio de Janeiro, Brazil; 3 Universidade Federal do Rio de Janeiro (UFRJ), Rio de Janeiro, Brazil; 4 Center for High Energy Physics, Tsinghua University, Beijing, China; 5 LAPP, Université de Savoie, CNRS/IN2P3, Annecy-Le-Vieux, France; 6 CNRS/IN2P3, LPC, Clermont Université, Université Blaise Pascal, Clermont-Ferrand, France; 7 CPPM, Aix-Marseille Université, CNRS/IN2P3, Marseille, France; 8 LAL, Université Paris-Sud, CNRS/IN2P3, Orsay, France; 9 LPNHE, Université Pierre et Marie Curie, Université Paris Diderot, CNRS/IN2P3, Paris, France; 10 Fakultät Physik, Technische Universität Dortmund, Dortmund, Germany; 11 Max-Planck-Institut für Kernphysik (MPIK), Heidelberg, Germany; 12 Physikalisches Institut, Ruprecht-Karls-Universität Heidelberg, Heidelberg, Germany; 13 School of Physics, University College Dublin, Dublin, Ireland; 14 Sezione INFN di Bari, Bari, Italy; 15 Sezione INFN di Bologna, Bologna, Italy; 16 Sezione INFN di Cagliari, Cagliari, Italy; 17 Sezione INFN di Ferrara, Ferrara, Italy; 18 Sezione INFN di Firenze, Firenze, Italy; 19 Laboratori Nazionali dell’INFN di Frascati, Frascati, Italy; 20 Sezione INFN di Genova, Genova, Italy; 21 Sezione INFN di Milano Bicocca, Milano, Italy; 22 Sezione INFN di Roma Tor Vergata, Roma, Italy; 23 Sezione INFN di Roma La Sapienza, Roma, Italy; 24 Henryk Niewodniczanski Institute of Nuclear Physics Polish Academy of Sciences, Kraków, Poland; 25 Faculty of Physics and Applied Computer Science, AGH - University of Science and Technology, Kraków, Poland; 26 National Center for Nuclear Research (NCBJ), Warsaw, Poland; 27 Horia Hulubei National Institute of Physics and Nuclear Engineering, Bucharest-Magurele, Romania; 28 Petersburg Nuclear Physics Institute (PNPI), Gatchina, Russia; 29 Institute of Theoretical and Experimental Physics (ITEP), Moscow, Russia; 30 Institute of Nuclear Physics, Moscow State University (SINP MSU), Moscow, Russia; 31 Institute for Nuclear Research of the Russian Academy of Sciences (INR RAN), Moscow, Russia; 32 Budker Institute of Nuclear Physics (SB RAS) and Novosibirsk State University, Novosibirsk, Russia; 33 Institute for High Energy Physics (IHEP), Protvino, Russia; 34 Universitat de Barcelona, Barcelona, Spain; 35 Universidad de Santiago de Compostela, Santiago de Compostela, Spain; 36 European Organization for Nuclear Research (CERN), Geneva, Switzerland; 37 Ecole Polytechnique Fédérale de Lausanne (EPFL), Lausanne, Switzerland; 38 Physik-Institut, Universität Zürich, Zürich, Switzerland; 39 Nikhef National Institute for Subatomic Physics, Amsterdam, The Netherlands; 40 Nikhef National Institute for Subatomic Physics and VU University Amsterdam, Amsterdam, The Netherlands; 41 NSC Kharkiv Institute of Physics and Technology (NSC KIPT), Kharkiv, Ukraine; 42 Institute for Nuclear Research of the National Academy of Sciences (KINR), Kyiv, Ukraine; 43 University of Birmingham, Birmingham, United Kingdom; 44 H.H. Wills Physics Laboratory, University of Bristol, Bristol, United Kingdom; 45 Cavendish Laboratory, University of Cambridge, Cambridge, United Kingdom; 46 Department of Physics, University of Warwick, Coventry, United Kingdom; 47 STFC Rutherford Appleton Laboratory, Didcot, United Kingdom; 48 School of Physics and Astronomy, University of Edinburgh, Edinburgh, United Kingdom; 49 School of Physics and Astronomy, University of Glasgow, Glasgow, United Kingdom; 50 Oliver Lodge Laboratory, University of Liverpool, Liverpool, United Kingdom; 51 Imperial College London, London, United Kingdom; 52 School of Physics and Astronomy, University of Manchester, Manchester, United Kingdom; 53 Department of Physics, University of Oxford, Oxford, United Kingdom; 54 Syracuse University, Syracuse, NY USA; 55 Pontifícia Universidade Católica do Rio de Janeiro (PUC-Rio), Rio de Janeiro, Brazil; 56 Institut für Physik, Universität Rostock, Rostock, Germany

## Abstract

The energy flow created in *pp* collisions at $\sqrt{s}=7\ \mbox{TeV}$ is studied within the pseudorapidity range 1.9<*η*<4.9 with data collected by the LHCb experiment. The measurements are performed for inclusive minimum-bias interactions, hard scattering processes and events with an enhanced or suppressed diffractive contribution. The results are compared to predictions given by Pythia-based and cosmic-ray event generators, which provide different models of soft hadronic interactions.

## Introduction

In Quantum Chromodynamics (QCD), the final state of an inelastic hadron-hadron collision can be described by contributions from hard and soft scattering occurring between the constituents of the hadrons, initial- and final-state (gluon) radiation and the fragmentation of the initially coloured partonic final state into colour-neutral hadrons. The soft component of a collision is called the underlying event. Its precise theoretical description remains a challenge, while the dynamics of hard scattering processes is well described by perturbative QCD. One source of the underlying event activity is multi-parton interactions (MPI). These arise mainly in the region of a very low parton momentum fraction, where parton densities are high so that the probability of more than a single parton-parton interaction per hadron-hadron collision is large. MPI effects become increasingly important at LHC collision energies, where inelastic interactions between very soft partons are sufficiently energetic to contribute to final state particle production [1].

MPI phenomena can be probed by measuring in the centre-of-mass system the amount of energy created in inelastic hadron-hadron interactions at large values of the pseudorapidity *η*=−ln[tan(*θ*/2)], with *θ* being the polar angle of particles with respect to the beam axis. The energy flow is expected to be directly sensitive to the amount of parton radiation and MPI [2]. For a particular pseudorapidity interval with width Δ*η*, the total energy flow, which is normalised to the number of inelastic *pp* interactions *N*_int_, is defined as

1 $$ \frac{1}{N_{\mathrm{int}}} \frac{dE_{\mathrm{total}}}{d\eta} = \frac{1}{\Delta\eta} \Biggl( \frac{1}{N_{\mathrm{int}}}\sum_{i=1}^{N_{\mathrm{part},\eta}}E_{i,\eta} \Biggr) , $$

where *N*_part,*η*_ is the total number of stable particles and *E*_*i*,*η*_ is the energy of the individual particles.

In this study, the energy flow is measured in *pp* collisions at $\sqrt{s}=7\ \mbox{TeV}$ within the pseudorapidity range 1.9<*η*<4.9. This extends the previous measurements that have been made in $p\bar{p}$ [3] and *ep* collisions [4] to larger pseudorapidity values and higher centre-of-mass energies, and complements the studies performed by the CMS and ATLAS collaborations [5, 6]. Experimental results are compared to predictions given by Pythia-based [7, 8] and cosmic-ray event generators [9, 10], which model the underlying event activity in different ways. In order to probe various aspects of multi-particle production in high-energy hadron-hadron collisions, the measurements are performed for the following four classes of events: inclusive minimum-bias, hard scattering, diffractive, and non-diffractive enriched interactions.

## The LHCb detector

The LHCb detector [11] is a single-arm forward spectrometer with an angular coverage from 10 mrad to 300 (250) mrad in the bending (non-bending) plane, designed for the study of *b*- and *c*-hadrons. The detector includes a high precision tracking system consisting of a silicon-strip vertex detector (VELO) surrounding the *pp* interaction region, a large-area silicon-strip detector located upstream of a dipole magnet with a bending power of about 4 Tm, and three stations of silicon-strip detectors and straw drift tubes placed downstream. The VELO has a larger angular acceptance than the rest of the spectrometer, including partial coverage of the backward region. It allows reconstruction of charged particle tracks in the pseudorapidity ranges 1.5<*η*<5.0 and −4<*η*<−1.5. The combined tracking system has a momentum resolution Δ*p*/*p* that varies from 0.4 % at 5 GeV/*c* to 0.6 % at 100 GeV/*c*, and an impact parameter resolution of 20 μm for tracks with high transverse momentum, *p*_T_. Charged hadrons are identified using two ring-imaging Cherenkov detectors. Photon, electron and hadron candidates are distinguished by a calorimeter system consisting of scintillating-pad and preshower detectors, an electromagnetic calorimeter (ECAL) and a hadronic calorimeter (HCAL). The calorimeters have an energy resolution of $\sigma(E)/E=10\ \%/\sqrt{E}\oplus 1\ \%$ and $\sigma(E)/E=69\ \%/\sqrt{E}\oplus 9\ \%$ (with *E* in GeV), respectively. Muons are identified by a system composed of alternating layers of iron and multiwire proportional chambers.

The trigger consists of a hardware stage, based on information from the calorimeter and muon systems, followed by a software stage which applies a full event reconstruction. For the minimum-bias data used in this analysis, the hardware trigger was accepting all beam–beam crossings, while the presence of at least one reconstructed track was required in the software stage to record an event.

## Data analysis

### Data and Monte Carlo samples

The analysis is performed using a sample of minimum-bias data collected in *pp* collisions at $\sqrt{s}=7\ \mbox{TeV}$ during the initial running period of the LHC with low interaction rate. The fraction of bunch crossings with two or more collisions (“pile-up events”) is estimated to be approximately 5 %. The total number of events available in the sample is 5.8×10^6^, corresponding to an integrated luminosity of about 0.1 nb^−1^.

Fully simulated minimum-bias *pp* events at $\sqrt{s}=7\ \mbox{TeV}$ were generated using the LHCb tune [12] of the Pythia 6.4 event generator [7]. Here, decays of hadronic particles are described by EvtGen [13] in which final state QED radiation is generated using Photos [14]. The interaction of the generated particles with the detector and its response are implemented using the Geant4 toolkit [15, 16] as described in Ref. [17]. Additional Monte Carlo (MC) samples with fully simulated minimum-bias *pp* interactions at $\sqrt{s}=7\ \mbox{TeV}$ were generated using the Perugia 0 and Perugia NOCR [18] tunes of Pythia 6.4. These models along with the LHCb tune use different values for the MPI energy scaling parameter and MPI *p*_T_ cut-off, which entails a sizeable deviation in the amount of MPI predicted by these tunes.

The LHCb tune utilises the CTEQ6L parton density functions (PDFs) [19], while both Perugia tunes use the CTEQ5L PDFs [20]. Colour reconnection effects are not included in the Perugia NOCR tune. In the MC samples generated with the Perugia 0 and Perugia NOCR tunes, diffractive *pp* interactions are not included, whereas the sample generated with the LHCb tune contains the contributions from both single and double diffractive processes. A sample of fully simulated diffractive events generated with Pythia 8.130 [8], which utilises the CTEQ5L PDFs, is used in addition. This event generator gives a more accurate description of diffractive *pp* interactions than Pythia 6, especially at high-*p*_T_, as it includes the contribution from hard diffractive processes, which is absent in Pythia 6 [21].

In addition to the models above, experimental results are compared to generator level predictions given by the Pythia 8.135 model with default parameters. Furthermore, the measurements are compared with predictions given by the cosmic-ray interaction models Epos 1.99 [22], Qgsjet01, QgsjetII-03 [23], and Sibyll 2.1 [24], which are widely used in extensive air shower simulations and are not tuned to LHC data. These generate inelastic *pp* interactions taking into account the contributions from both soft and hard scattering processes. Soft contributions are described with Gribov’s Reggeon field theory [25] via exchanges between virtual quasi-particle states (Pomerons), while hard processes are described by perturbative QCD via exchanges of hard or semi-hard Pomerons. The predictions given by these models diverge mainly because of different treatments of non-linear interaction effects related to parton saturation [26] and shadowing [27]. The Qgsjet01 model describes hadronic multiple scattering processes as multiple exchanges of Pomerons without specific treatment of saturation effects. A distinct feature of the QgsjetII model is the treatment of non-linear parton effects via Pomeron interactions taking into account all order re-summation of the corresponding Reggeon field theory diagrams. Based on the dual parton model [28], Sibyll utilises the Lund string model [29] for hadronisation and describes soft and hard processes using the Pomeron formalism and the minijet model [30], correspondingly. The treatment of non-linear effects in this model is based on a simple geometrical approach of parton saturation. The Epos model takes into account energy-momentum correlations between multiple re-scatterings and describes non-linear effects using an effective treatment of lowest order Pomeron–Pomeron interaction graphs. It also accounts for the final state interaction of the produced particles.

### Analysis strategy

The energy flow, as defined in Eq. (1), is the energy-weighted pseudorapidity distribution of particles, normalised to the number of inelastic interactions and the *η*-bin size. The measurements are performed in ten equidistant pseudorapidity bins of width Δ*η*=0.3 over the range 1.9<*η*<4.9. The primary measurement is the energy flow carried by charged particles (charged energy flow). It is performed with reconstructed tracks which contain hits in the VELO and downstream tracking stations and have momentum in the range 2<*p*<1000 GeV/*c*. Particle identification is not required in this analysis, as the energy is taken from the momentum, neglecting particle masses. In order to be able to compare the results of the measurements with generator level predictions, the reconstructed charged energy flow is corrected for detector effects. The total energy flow is determined by using a data-constrained MC estimate of the neutral component based on information from the ECAL, while the HCAL is not used. Details of the procedure are discussed below.

### Event classes

The event classes studied in this analysis are defined as follows. Inclusive minimum-bias events are selected by requesting the presence of at least one track originating from the luminous region in order to suppress pollution from beam–gas interactions and beam halo related background. Events with two or more reconstructed primary vertices are rejected to suppress pile-up contamination. To minimise biases on the track multiplicity of the event, the information on the primary vertex is not used. The selected inclusive minimum-bias interactions are further classified as hard scattering, diffractive and non-diffractive enriched events using the following criteria:

- Hard scattering events: at least one track with *p*_T_>3 GeV/*c* and 1.9<*η*<4.9.
- Diffractive enriched events: no tracks reconstructed with −3.5<*η*<−1.5.
- Non-diffractive enriched events: at least one track reconstructed with −3.5<*η*<−1.5.

The selection requirements applied for the last two event classes are motivated by the fact that a sizeable rapidity gap is an experimental signature of diffractive processes [31]. The level of enrichment of the diffractive and non-diffractive samples was studied in simulation, by retrieving the Pythia process type of the *pp* interaction for every selected diffractive and non-diffractive candidate. In the case of the LHCb tune of Pythia 6.4, the purities of the selected diffractive and non-diffractive enriched samples are found to be about 70 % and 90 %, respectively. Although the actual percentages are only meaningful within the specific model, the study shows that the applied selection criteria indeed lead to sizeable enhancement of the respective event classes.

To minimise the experimental corrections, the definition of the event classes at generator level is similar to that at detector level. Inclusive minimum-bias events at generator level are selected by requiring the presence of at least one outgoing final-state charged particle (lifetime *τ*>10^−8^ s) in the pseudorapidity range 1.9<*η*<4.9, but without imposing any condition on its energy. The sample of hard scattering events is selected by requesting at least one final-state charged particle with *p*_T_>3 GeV/*c* and 1.9<*η*<4.9. The absence or presence of at least one final-state charged particle in −3.5<*η*<−1.5 is used as criterion to select diffractive and non-diffractive enriched events among inclusive minimum-bias interactions, respectively. For the selected events, the energy flow at generator level is determined using the outgoing final-state charged and neutral particles^1^ which are either prompt, originating directly from the fragmentation, or the decay products of unstable particles. Since neutrinos are not reconstructed by the LHCb spectrometer the energy carried by these particles is not taken into account. Only MC events simulated with exactly one inelastic *pp* interaction are considered in this study.

### Corrections

The reconstructed charged energy flow measured with data is corrected for detector effects using bin-by-bin correction factors, which are estimated as the ratio of the charged energy flow at generator and detector level in simulation for each *η* region and event class under consideration. The overall correction factor for each bin is taken as the average of the correction factors obtained with different MC models used in this analysis. For inclusive, hard scattering and non-diffractive enriched events, the average and standard deviation of the correction factors, which is included in the model-dependent systematic uncertainty, are determined from the LHCb, Perugia 0 and Perugia NOCR tunes of Pythia 6.4. In the case of the diffractive enriched event class, only the LHCb tune and the Pythia 8 diffractive simulation are used. Except for the lowest *η* bin, which suffers from reduced acceptance for low-*p*_T_ particles and thus exhibits large corrections and a sizeable model dependence, the correction factors are found to be stable among the models with a slight rise towards the edges of the detector acceptance. The majority of the factors are well below two, indicating that most of the energy is measured by the detector. In the case of diffractive enriched events, the correction factors obtained with the LHCb tune are slightly smaller than unity for some of the bins, i.e. the energy flow at detector level is found to be larger than at generator level. This is due to detection inefficiency for charged particles over the pseudorapidity range −3.5<*η*<−1.5. As a result, some of the events containing backward going charged particles migrate into the diffractive sample, which leads to enhanced energy flow at detector level.

For the measurement of the total energy flow, the neutral component *F*_neut,*η*_ is estimated in the following way. To first order *F*_neut,*η*_ is assumed to be proportional to the corrected charged energy flow *F*_char,*η*_ with a factor *R*_gen,*η*_, which is the average ratio of the neutral energy flow to the charged energy flow obtained at generator level for each *η* bin and event class with different Pythia tunes. This ratio is found to be rather stable over the entire pseudorapidity range of the measurements with only small variations between the Pythia tunes. This reflects the usage of the same hadronisation mechanism governed in the Pythia generator by the Lund string model [7, 8]. The latter successfully describes the hadronisation of quarks and gluons emerging from high energy interactions and was rigorously tested for high-*p*_T_ processes [32–34]. The *R*_gen,*η*_ ratio is found to be around 0.6 for all event types except the hard scattering interactions. For the latter, it is about 15 % smaller for all *η* bins. This feature is found to be a consequence of the requirement of a high-*p*_T_ charged particle in the definition of this event class.

Under the assumption outlined above, the total energy flow for a particular event class and pseudorapidity bin *F*_total,*η*_ can be written as

2 $$ F_{\mathrm{total},\eta} = F_{\mathrm{char},\eta}+F_{\mathrm{neut},\eta} = F_{\mathrm{char},\eta} \times (1+R_{\mathrm{gen},\eta} ). $$

In order to constrain this initially purely model-based estimate of the neutral energy flow to data, the total energy flow is further multiplied by an additional correction factor *k*_*η*_. It accounts for differences between simulation and data being defined for every *η* bin as

3 $$ k_{\eta} = \frac{1+R_{\mathrm{data},\eta}}{1+R_{\mathrm{mc},\eta}} . $$

Here, *R*_data,*η*_ and *R*_mc,*η*_ are ratios of the uncorrected neutral to charged energy flow measured in data and simulation, respectively. The *R*_mc,*η*_ ratio is obtained with different Pythia tunes and its average is taken for the estimation of the *k*_*η*_ factors. The neutral component of *R*_data,*η*_ and *R*_mc,*η*_ is measured using reconstructed photon candidates which are selected from neutral clusters in the ECAL with an energy greater than 2 GeV and *p*_T_>0.2 GeV/*c*. Since the polar angular coverage of the ECAL begins at about 30 mrad, there are no measurements of the neutral energy for the last two *η* bins (*η*>4.3). The *k*_*η*_ factors for this pseudorapidity region are estimated using a linear extrapolation of the *k*_*η*_ factors obtained for the pseudorapidity interval 3.1<*η*<4.3. The bins with *η*<3.1 are not considered for the extrapolation, as these are affected by the detection inefficiency for low-*p*_T_ charged particles. The latter have a low average momentum in this *η* region and thus are unlikely to reach downstream tracking stations. Except for the lowest *η* bin, which suffers most from the detection inefficiency especially in the case of the diffractive enriched event class, the *k*_*η*_ factors are found to be rather close to unity, reflecting the fact that the ratio of the neutral to charged energy flow is well simulated at detector level.

## Systematic uncertainties

The total uncertainties on the results are dominated by systematic effects, as the statistical uncertainties are found to be negligible for all *η* bins and event classes. The various contributions to the systematic uncertainties are summarised in Table 1.

**Table 1:** Relative systematic uncertainties (in percent) affecting the energy flow measurements for all event classes. The total uncertainties are obtained by adding the individual sources in quadrature. The ranges indicate the variation of the uncertainty as a function of *η*

Source of uncertainty	Inclusive minbias	Hard scattering	Diffractive enriched	Non-diffractive enriched
Model uncertainty on correction factors	0.6–9.2	0.7–4.1	16–43	0.7–8.6
Selection cuts	1.0–4.9	2.7–8.8	0.9–2.8	1.1–5.0
Tracking efficiency	3	3	3	3
Multiple tracks	1	1	1	1
Spurious tracks	0.3–1.2	0.4–1.7	0.2–0.7	0.3–1.2
Magnet polarity	–	–	2.6–7.7	–
Residual pile-up	1.7	1.7	1.7	1.7
Total on *F*_char,*η*_	3.9–11	4.9–10	16–43	4.0–11
Variation of *R*_gen,*η*_ and *k*_*η*_ factors	0.8–6.1	0.7–2.9	1.5–23	0.9–5.5
Photon efficiency	1.4–1.6	1.2–1.3	1.3–2.3	1.3–1.6
ECAL miscalibration	<1	<1	<1	<1
Total on *F*_total,*η*_	4.4–13	5.4–11	17–49	4.4–12

For all event types except hard scattering interactions, the largest uncertainty on the charged energy flow arises from the model dependence of the bin-by-bin correction factors, which is estimated as the standard deviation of the correction factors obtained with different Pythia tunes. Here, the largest impact is at low *η*, reaching 9 % for inclusive and non-diffractive enriched events, 4 % for hard scattering interactions and up to 43 % for diffractive enriched events. At large *η* this effect generally drops to about 1–2 % for all event classes except diffractive enriched interactions for which it stays above 15 %.

Systematic uncertainties related to the track selection requirements are estimated by comparing the fraction of the energy flow from tracks which are rejected by the selection cuts in data and simulation. For the majority of the bins the resulting systematic uncertainty is found to be less than 4 %. Only for hard scattering events this uncertainty approaches 9 % at low *η*.

To account for differences between the true tracking efficiency and that estimated using simulation, a global 3 % systematic uncertainty is assigned across the entire *η* range following the analysis presented in Ref. [35]. This applies for all event classes under consideration.

The other tracking related factors having an influence on the charged energy flow measurements are contaminations from multiply reconstructed tracks and tracks created from random combinations of hits (spurious tracks). The impact of the former is estimated by removing from the measurement all tracks found within the same event with similar momentum vectors. It is observed that the charged energy flow drops by less than 1 % for all *η* bins and event classes in case of both data and simulation. For the final results, a global 1 % systematic uncertainty for multiply reconstructed tracks is conservatively assigned. The effect of spurious tracks is estimated in simulation by determining the energy flow carried by reconstructed tracks which cannot be associated with particles at generator level and accounting for the difference between the rate of spurious tracks in data and simulation. The corresponding systematic uncertainty is found to vary between 0.2 % and 2 %.

It has been checked that reversing the LHCb magnet polarity has only an influence on the measurements of the charged energy flow for the diffractive enriched event class, which mainly consists of low-multiplicity events. Here, the corresponding effect is assigned as a systematic uncertainty.

Events with more than one reconstructed primary vertex are vetoed in the analysis in order to suppress pile-up contamination. Its residual effect is estimated to be 1.7 % by taking the efficiencies to accept pile-up events from simulation. This factor is included in the normalisation of the energy flow and conservatively taken as the systematic uncertainty.

The total energy flow acquires an additional uncertainty from the variation of the *R*_gen,*η*_ and *k*_*η*_ factors between the Pythia tunes and the extrapolation procedure used for the *k*_*η*_ factors in two highest *η* bins. No systematic uncertainty is assigned to account for inaccuracies of the Lund string model in describing the ratio of the neutral energy flow to the charged energy flow. The uncertainties associated with the ECAL energy calibration and photon reconstruction efficiency also affect the accuracy of the total energy flow. To account for a possible difference in the photon reconstruction efficiency between simulation and data, a global 3.7 % systematic uncertainty is assigned following the analysis presented in Ref. [36]. The energy calibration of the ECAL has been studied by measuring the invariant masses of diphoton resonances (*π* ^0^→*γγ* and *η*→*γγ*) and has been assigned a global systematic uncertainty of 1.5 %. Both uncertainties are scaled with a factor *F*_neut,*η*_/*F*_total,*η*_ and are listed in Table 1.

Other potential sources of systematic uncertainties such as momentum- and *η*-smearing, effect of the beam crossing angle, neglecting the masses of charged particles, pollution from elastic scattering and beam-gas interactions have been studied as well. Their impacts on the accuracy of the measurements are found to be negligible.

The total systematic uncertainties on the corrected charged and total energy flow are listed in Tables 2 and 3 for all event classes and *η* bins. It should be noted that the uncertainties are strongly correlated between the bins.

**Table 2:** Charged energy flow for all event classes and *η* bins with the corresponding systematic uncertainties. The statistical uncertainties are insignificant and not listed. All values are in GeV per unit pseudorapidity interval

Pseudorapidity range	Inclusive minbias	Hard scattering	Diffractive enriched	Non-diffractive enriched
1.9<*η*<2.2	12 ± 1	37 ± 4	4 ± 2	13 ± 1
2.2<*η*<2.5	16 ± 1	50 ± 4	5 ± 2	17 ± 1
2.5<*η*<2.8	21 ± 1	64 ± 4	6 ± 2	22 ± 1
2.8<*η*<3.1	27 ± 1	83 ± 5	9 ± 3	29 ± 1
3.1<*η*<3.4	35 ± 2	105 ± 6	12 ± 3	38 ± 2
3.4<*η*<3.7	46 ± 2	132 ± 6	17 ± 4	49 ± 2
3.7<*η*<4.0	58 ± 2	161 ± 8	22 ± 5	61 ± 2
4.0<*η*<4.3	73 ± 3	194 ± 10	31 ± 7	77 ± 3
4.3<*η*<4.6	88 ± 4	219 ± 12	41 ± 7	93 ± 4
4.6<*η*<4.9	112 ± 5	256 ± 13	57 ± 9	118 ± 6

**Table 3:** Total energy flow for all event classes and *η* bins with the corresponding systematic uncertainties. The statistical uncertainties are insignificant and not listed. All values are in GeV per unit pseudorapidity interval

Pseudorapidity range	Inclusive minbias	Hard scattering	Diffractive enriched	Non-diffractive enriched
1.9<*η*<2.2	18 ± 2	55 ± 6	4 ± 2	19 ± 2
2.2<*η*<2.5	26 ± 2	77 ± 7	6 ± 2	28 ± 2
2.5<*η*<2.8	36 ± 2	102 ± 7	10 ± 3	38 ± 2
2.8<*η*<3.1	48 ± 3	133 ± 8	15 ± 5	51 ± 3
3.1<*η*<3.4	60 ± 3	164 ± 9	20 ± 5	64 ± 3
3.4<*η*<3.7	75 ± 3	203 ± 11	27 ± 6	80 ± 4
3.7<*η*<4.0	95 ± 4	246 ± 15	37 ± 9	100 ± 4
4.0<*η*<4.3	118 ± 5	296 ± 17	50 ± 11	125 ± 6
4.3<*η*<4.6	144 ± 7	329 ± 20	65 ± 11	151 ± 7
4.6<*η*<4.9	182 ± 9	380 ± 21	89 ± 15	191 ± 10

## Results

**Fig. 1:**
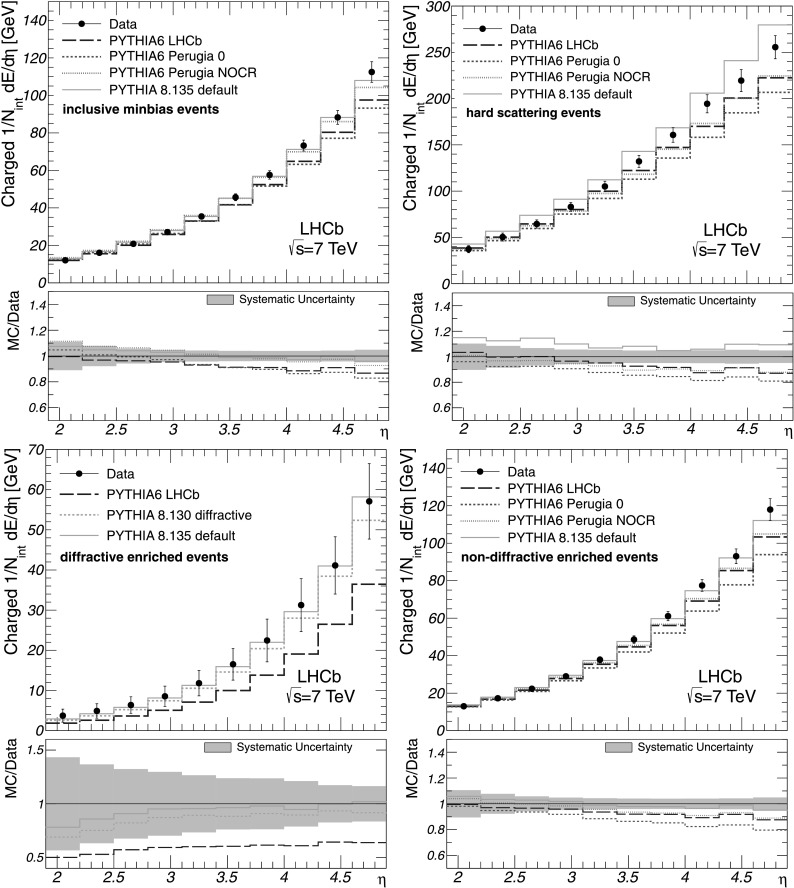
Charged energy flow as a function of *η* for all event classes as indicated in the figures. The corrected measurements are given by *points* with error bars, while the predictions by the Pythia tunes are shown as histograms. The error bars represent the systematic uncertainties, which are highly correlated between the bins. The statistical uncertainties are negligible. The ratios of MC predictions to data are shown in addition

The fully-corrected measurements for the charged energy flow are shown in Fig. 1 for each event class together with the generator level predictions given by the Pythia tunes and the corresponding systematic uncertainties. By comparing experimental results obtained for different event classes one can clearly see that the amount of energy flow strongly correlates with the momentum transfer in an underlying *pp* inelastic interaction. The charged energy flow rises more steeply with pseudorapidity in data than predicted by the majority of the Pythia tunes. As a consequence, the discrepancy between the measurements and generator level predictions increases towards large *η* rising to 20 % in the last *η* bin. At lower *η* the data are reasonably well described by the Pythia tunes. This is the case for all event classes except the diffractive enriched one. For the latter, the measurements are well described by the Pythia 8.135 generator with default parameters. However, this model overestimates the charged energy flow in the case of hard scattering events over the entire pseudorapidity range of the measurements.

**Fig. 2:**
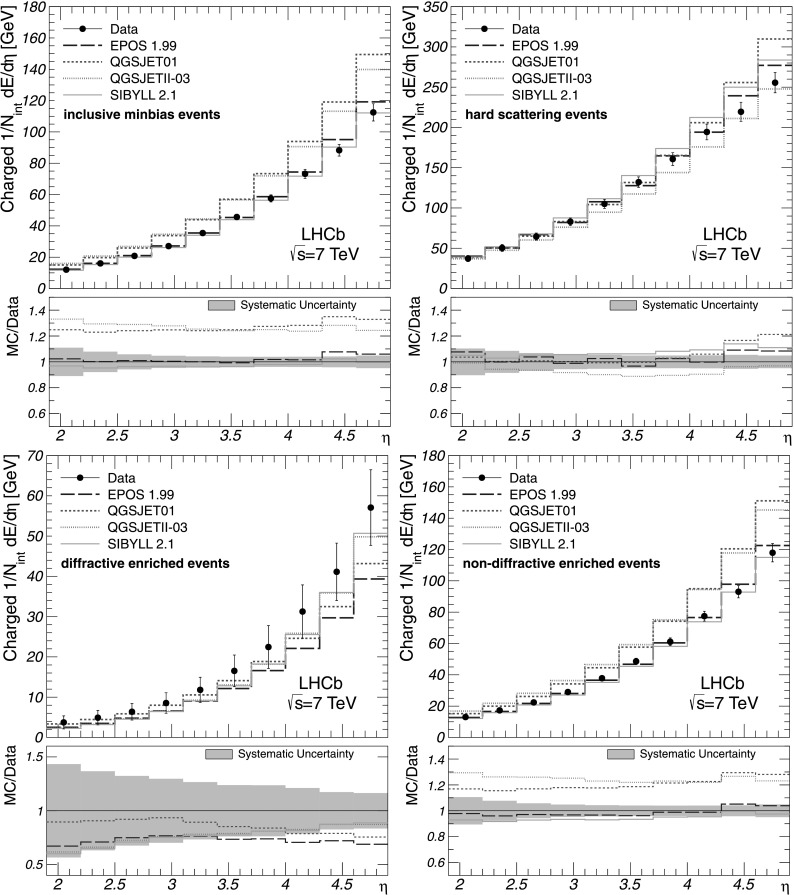
Charged energy flow as a function of *η* for all event classes as indicated in the figures. The corrected measurements are given by *points* with error bars, while the predictions by the cosmic-ray interaction models are shown as histograms. The error bars represent the systematic uncertainties, which are highly correlated between the bins. The statistical uncertainties are negligible. The ratios of MC predictions to data are shown in addition

Figure 2 illustrates the charged energy flow along with the predictions given by the cosmic-ray interaction models. It is interesting to note that the measurements performed with inclusive minimum-bias and non-diffractive enriched events are well described by the Epos 1.99 and Sibyll 2.1 models, while the Qgsjet01 and QgsjetII-03 generators overestimate the charged energy flow for these event classes. The latter also occurs at large *η* in the case of hard scattering interactions for all cosmic-ray interaction models except the QgsjetII-03. The diffractive enriched charged energy flow is underestimated by all cosmic-ray interaction models.

**Fig. 3:**
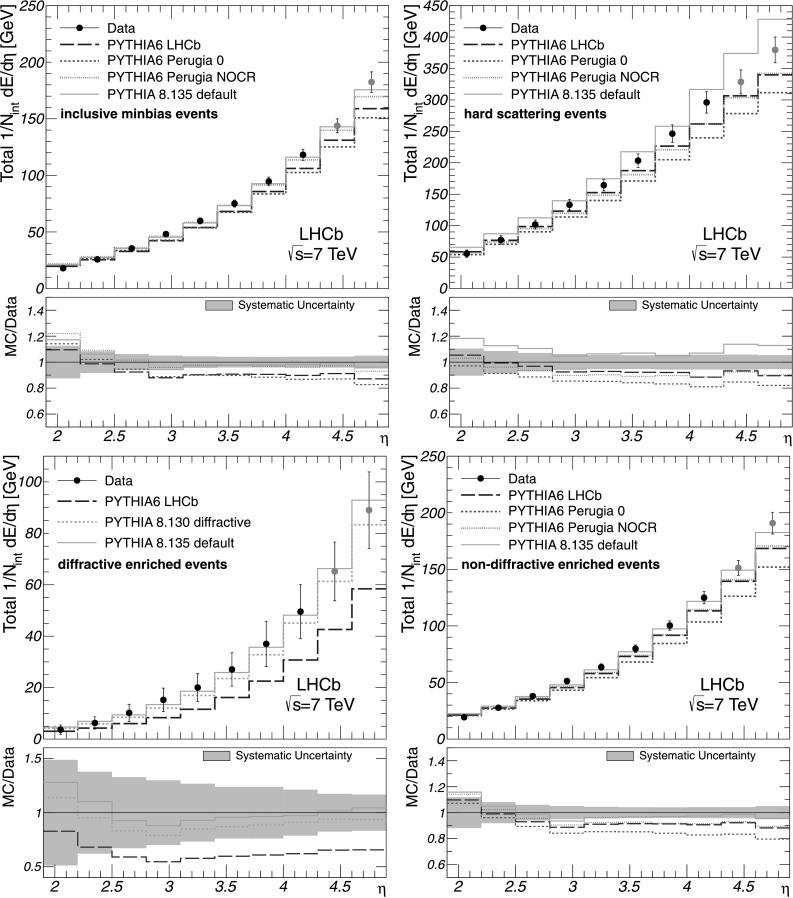
Total energy flow as a function of *η* for all event classes as indicated in the figures. The corrected measurements are given by *points* with error bars, while the predictions by the Pythia tunes are shown as histograms. The data obtained with extrapolated *k*_*η*_ factors are shown in *grey*. The error bars represent the systematic uncertainties, which are highly correlated between the bins. The statistical uncertainties are negligible. The ratios of MC predictions to data are shown in addition

The total energy flow is shown for each event class in Fig. 3 along with the generator level predictions given by the Pythia tunes and the corresponding systematic uncertainties. It can be clearly seen that all Pythia 6 tunes underestimate the amount of energy flow at large pseudorapidity for all event classes. The Pythia 8.135 generator gives the best description of the measurements performed with inclusive minimum-bias, diffractive and non-diffractive enriched events among the Pythia tunes, except for the pseudorapidity range 1.9<*η*<2.5. None of these models provide an accurate description of the energy flow measured with hard scattering events. The predictions given by the LHCb and Perugia NOCR tunes for non-diffractive enriched and hard scattering events are rather similar, while the Perugia 0 tune significantly underestimates the energy flow for all event classes. For diffractive enriched events, the inconsistency between the data and the prediction given by the LHCb tune is found to be rather large throughout the entire pseudorapidity range 1.9<*η*<4.9, while the Pythia 8.135 generator with default parameters gives a good description of the corresponding energy flow at large *η*.

**Fig. 4:**
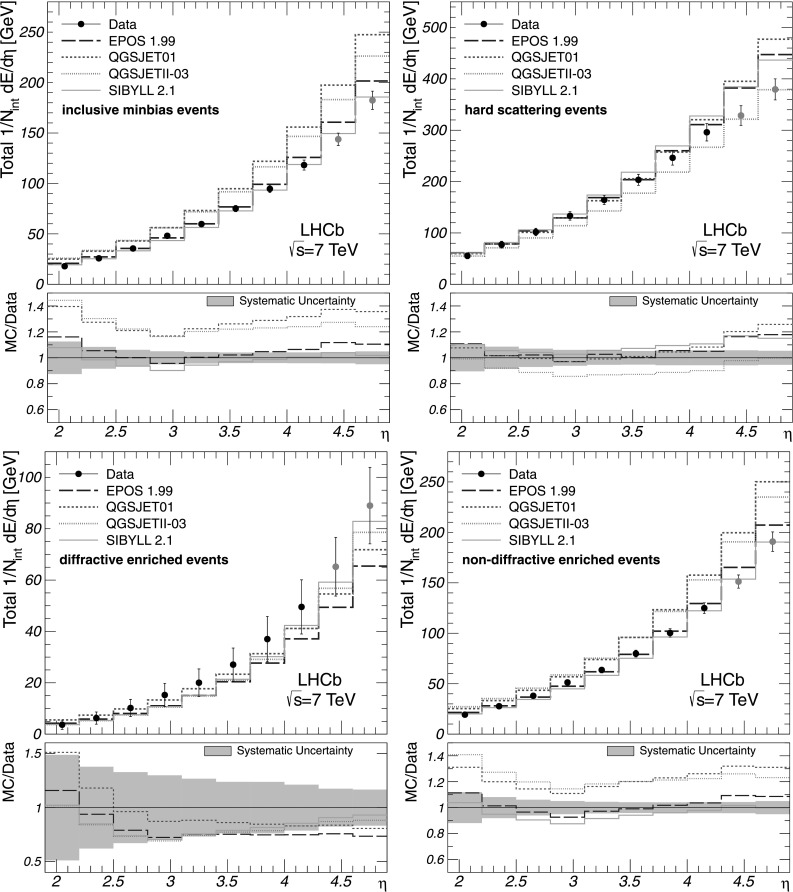
Total energy flow as a function of *η* for all event classes as indicated in the figures. The corrected measurements are given by *points* with error bars, while the predictions by the cosmic-ray interaction models are shown as histograms. The data obtained with extrapolated *k*_*η*_ factors are shown in *grey*. The error bars represent the systematic uncertainties, which are highly correlated between the bins. The statistical uncertainties are negligible. The ratios of MC predictions to data are shown in addition

Figure 4 illustrates the total energy flow together with the predictions given by the cosmic-ray interaction models. It is observed that the Sibyll 2.1 generator gives the best description of the energy flow measured with inclusive minimum-bias and non-diffractive enriched events at large *η*. The predictions given by the Epos 1.99 generator for these event classes also describe the measurements reasonably well. In the case of hard scattering interactions, the best description of the data at large *η* is given by the QgsjetII-03 generator. The total energy flow measured with diffractive enriched events is underestimated at large *η* by all cosmic-ray interaction models used in this study. The measurements of the charged and total energy flow are summarised in Tables 2 and 3 for all event classes and *η* bins.

The results obtained in this study cannot be directly compared with the measurements performed by the CMS collaboration [5], since different event selection criteria are applied in the analyses. Nevertheless, both measurements demonstrate that the energy flow is underestimated by Pythia 6 tunes at large pseudorapidity, while the results of the ATLAS collaboration indicate that the amount of transverse energy is also underestimated by various Pythia tunes at large *η* [6].

## Conclusions

The energy flow is measured in the pseudorapidity range 1.9<*η*<4.9 with data collected by the LHCb experiment in *pp* collisions at $\sqrt{s}=7\ \mbox{TeV}$ for inclusive minimum-bias interactions, hard scattering processes and events with enhanced or suppressed diffractive contribution. The primary measurement is the energy flow carried by charged particles. For the measurement of the total energy flow, a data-constrained MC estimate of the neutral component is used. The energy flow is found to increase with the momentum transfer in an underlying *pp* inelastic interaction. The evolution of the energy flow as a function of pseudorapidity is reasonably well reproduced by the MC models. Nevertheless, the majority of the Pythia tunes underestimate the measurements at large pseudorapidity, while most of the cosmic-ray interaction models overestimate them, except for diffractive enriched interactions. For inclusive and non-diffractive enriched events, the best description of the data at large *η* is given by the Sibyll 2.1 and Pythia 8.135 generators. The latter also provides a good description of the energy flow measured with diffractive enriched events, especially at large *η*. The comparison shows that the absence of hard diffractive processes moderates the amount of the forward energy flow meaning that their inclusion is vital for a more precise description of partonic interactions. It also demonstrates that higher-order QCD effects as contained in the Pomeron phenomenology play an important role in the forward region. None of the event generators used in this analysis are able to describe the energy flow measurements for all event classes that have been studied.

## Footnotes

1 These include *π* ^±^, *K* ^±^, *e* ^±^, *μ* ^±^, *p*, ${\overline{{ {{p}}}}}$, *γ*, *n*, ${\overline{{ {{n}}}}}$ and ${ { {{K}}}^{0}_{\mathrm{L}}}$.
